# Identification and Validation of a Novel DNA Damage and DNA Repair Related Genes Based Signature for Colon Cancer Prognosis

**DOI:** 10.3389/fgene.2021.635863

**Published:** 2021-02-24

**Authors:** Xue-quan Wang, Shi-wen Xu, Wei Wang, Song-zhe Piao, Xin-li Mao, Xian-bin Zhou, Yi Wang, Wei-dan Wu, Li-ping Ye, Shao-wei Li

**Affiliations:** ^1^Laboratory of Cellular and Molecular Radiation Oncology, Department of Radiation Oncology, Radiation Oncology Institute of Enze Medical Health Academy, Affiliated Taizhou Hospital of Wenzhou Medical University, Taizhou, China; ^2^Key Laboratory of Minimally Invasive Techniques & Rapid Rehabilitation of Digestive System Tumor of Zhejiang Province, Linhai, China; ^3^Wenzhou Medical University, Wenzhou, China; ^4^Department of Urology, Taizhou Hospital of Zhejiang Province Affiliated to Wenzhou Medical University, Linhai, China; ^5^Department of Gastroenterology, Taizhou Hospital of Zhejiang Province Affiliated to Wenzhou Medical University, Linhai, China

**Keywords:** mRNA signature, DNA damage, DNA repair, prediction, prognosis, colon cancer

## Abstract

**Backgrounds:** Colorectal cancer (CRC) with high incidence, has the third highest mortality of tumors. DNA damage and repair influence a variety of tumors. However, the role of these genes in colon cancer prognosis has been less systematically investigated. Here, we aim to establish a corresponding prognostic signature providing new therapeutic opportunities for CRC.

**Method:** After related genes were collected from GSEA, univariate Cox regression was performed to evaluate each gene’s prognostic relevance through the TCGA-COAD dataset. Stepwise COX regression was used to establish a risk prediction model through the training sets randomly separated from the TCGA cohort and validated in the remaining testing sets and two GEO datasets (GSE17538 and GSE38832). A 12-DNA-damage-and-repair-related gene-based signature able to classify COAD patients into high and low-risk groups was developed. The predictive ability of the risk model or nomogram were evaluated by different bioinformatics‐ methods. Gene functional enrichment analysis was performed to analyze the co-expressed genes of the risk-based genes.

**Result:** A 12-gene based prognostic signature established within 160 significant survival-related genes from DNA damage and repair related gene sets performed well with an AUC of ROC 0.80 for 5 years in the TCGA-CODA dataset. The signature includes CCNB3, ISY1, CDC25C, SMC1B, MC1R, LSP1P4, RIN2, TPM1, ELL3, POLG, CD36, and NEK4. Kaplan-Meier survival curves showed that the prognosis of the risk status owns more significant differences than T, M, N, and stage prognostic parameters. A nomogram was constructed by LASSO regression analysis with T, M, N, age, and risk as prognostic parameters. ROC curve, C-index, Calibration analysis, and Decision Curve Analysis showed the risk module and nomogram performed best in years 1, 3, and 5. KEGG, GO, and GSEA enrichment analyses suggest the risk involved in a variety of important biological processes and well-known cancer-related pathways. These differences may be the key factors affecting the final prognosis.

**Conclusion:** The established gene signature for CRC prognosis provides a new molecular tool for clinical evaluation of prognosis, individualized diagnosis, and treatment. Therapies based on targeted DNA damage and repair mechanisms may formulate more sensitive and potential chemotherapy regimens, thereby expanding treatment options and potentially improving the clinical outcome of CRC patients.

## Introduction

Colon cancer is a malignant intestinal disease with the highest incidence among gastrointestinal diseases. Colorectal cancer is the third most common cancer and one of the major cancers for mortality all over the world (Bray et al., 2018). The application of combined drugs, including adjuvant chemotherapy and radiotherapy (Dekker and Rex, 2018), is currently a worldwide accepted standard treatment for colon cancer. Besides, early diagnosis of primary or recurrent colon cancer is one of the key factors for the prognosis. Unfortunately, how to diagnose colon cancer early remains one of the most difficult issues in cancer treatment. The study reported in-depth research on the diagnosis and treatment of colon cancer, such as endoscopic diagnosis (Dekker and Rex, 2018), tumor markers (Sveen et al., 2020), and molecular targeted therapy (Ganesh et al., 2019). The American Joint Committee on Cancer divided the patients into stages I, IIa, IIb, IIIa, IIIb, IIIc, and IV according to the tumor-node-metastasis (TNM). The TNM staging can distinguish patients with different prognoses (O’Connell et al., 2004). There is still a possibility of recurrence in stage I to III patients who underwent curative resection, and the likelihood of recurrence increases with time and stage. However, due to complex pathogenesis and high metastasis rate, the diagnosis is still unsatisfactory, and the prognosis is poor (Kobayashi et al., 2007). Therefore, there is an urgent need to identify new diagnostic and prognostic biomarkers, therapeutic targets, and look into the potential molecular mechanisms of CRC. Today, the revolution helps to identify disease-related biomarkers through more novel bioinformatics analysis and the use of next-generation sequencing technology (Moody et al., 2017), which will help the early identification of colon cancer and the development of personalized treatment plans to benefit more patients.

There is an increasing interest in the search for new genes and the construction of multi-gene prediction models recently. Genome analysis based on the TCGA network project containing 276 patients’ CRC samples and corresponding germline DNA samples showed that some genes have been shown to be associated with highly mutated CRC (Ganesh et al., 2019). In hypermutated cancers, APC, TGFBR2, BRAF, MSH3, MSH6, SLC9A9, and TCF7L2 were highly mutated, in particular the frequent mutations of BRAF (V600E). On the contrary, the mutation rate of TP53 and APC was lower. In non-hypermutated cancer, APC, TP53, KRAS, PIK3CA, FBXW7, SMAD4, and NRAS were frequently mutated. Based on the mutation status, CRC could be divided into the non-hypermutated group (84%) and the hypermutated group (16%; Moody et al., 2017). Different studies have identified that CDX2, LC3B, ULBP2, SEMA5A, VEGF-D, and SMAD7 are potential biomarkers for the prognosis of colon cancer (Lord and Ashworth, 2017; Gourley et al., 2019; Mauri et al., 2020). However, the prognostic value of a single-gene related clinical prognostic model for CRC patients based on these genes is still not ideal. Yang et al. have constructed a 20 gene signature based on the expression profile of GSE44076 about colon cancer, which were considered as diagnosis targets for colon cancer (Chen et al., 2014).

In recent years, research on new therapeutic targets for different cancer types has gradually focused on genomic changes in the DNA damage response (DDR) pathway (Mauri et al., 2020). The current research on anti-tumor drugs mainly focuses on two main types: Platinum compounds and poly ADP-ribose polymerase inhibitors (Lord and Ashworth, 2017; Gourley et al., 2019). DDR changes were originally found in breast cancer and ovarian cancer, while it has now expanded to prostate and pancreatic cancer (Mauri et al., 2020). The role of DDR alterations in colorectal cancer is still not fully studied. There are only a few studies on its clinical impact and no orderly study system has been established (Chen et al., 2014; Lei et al., 2019; Sun et al., 2019; Karpov et al., 2020; Mauri et al., 2020; Scagliarini et al., 2020; Yu et al., 2020).

In our study, we aimed to construct a DNA damage and repair related gene-based signature and nomogram to make an improvement on the prognostic value of CRC through comprehensive bioinformatics methods.

## Materials and Methods

### Data Collection

The DNA damage and DNA repair related genes list were collected from GSEA gene sets^1^ by the keyword “DNA AND damage” or “DNA AND repair.” At last, 1545 genes related to DNA damage and repair were included in the analysis (Supplementary Table 1).

The gene expression data of HTseq RNA profiles FPKM (fragments per kilobase of exon per million reads mapped) of 471 COAD and 41 compared normal samples were extracted from The Cancer Genome Atlas-Colon adenocarcinoma (TCGA-COAD).^2^ Survival endpoint (vital status, days to the last follow-up, and days to death), age, stage, and histological type of primary of each patient were also retrieved.

The public expression profiles data of colon cancer were extracted from the GEO database^3^ by the keywords [“Colonic Neoplasms” (MeSH)]. The selected data must meet the following inclusion criteria: human gene expression profiles data of solid tissues of colon cancer, the datasets contained prognosis survival information, and enough samples for analysis. Four eligible data (GSE17538 and GSE38832, GSE44861 and GSE44076), based on the platform of Affymetrix-GPL570, Affymetrix-GPL570, Affymetrix-GPL, and Affymetrix-GPL13667 respectively, that met the above criteria were annotated based on the annotation platform and enrolled in this study, each GEO data set was checked the gene expression distribution was through the histogram and normalization. Furthermore, the related clinical data of the four datasets were retrieved.

### Construction of the DNA Damage and DNA Repair Related Gene Signature

All analyses in this study conducted in R language used R version 4.03. Univariate Cox regression analysis (Cox, 1972) was first performed with DNA damage and DNA repair related genes, and genes with a *p* value of less than 0.05 were considered a statistically significant difference. After randomly separating samples into the training set and testing set, genes that were strongly associated with OS of COAD patients were used for multivariate Cox hazards regression base based on the training set with the stepwise method in My.stepwise package (Hu, 2017). The process and results are shown in the Supplementary Material. Then a multivariate cox hazards regression model was built to assess the prognostic value for COAD.

The hazards model was established by the selected final gene signature, and the risk score was generated according to the following formula:

$$Riskscore=\overset{N}{\underset{i=1}{\sum }}\beta i*Ei$$

(N represents the total number of signature genes, *β*i and Ei represent the coefficient index, and the gene expression level of each gene, respectively)

Based on the risk score of each patient, samples were grouped into high risk and low-risk groups based on the risk score of each patient, and the relationship between risk and clinical data was then investigated.

### The Nomogram Establishing

All clinical prognostic factors T, M, N, age, and stage together with risk group were used for the selection of the prognostic parameters by Least Absolute Shrinkage and Selection Operator (LASSO; Friedman et al., 2010) regression analysis. And a related prognostic nomogram to assess the probability of 0.5-, 1-, and 3-year OS for COAD patients were built by “rms” R package. Calibration plots were used to evaluate the discriminative ability of the nomogram.

### Validation of the Multi-Gene Prognostic Signature

Firstly, survival analysis between high and low groups combined with clinical stage and the histological type was evaluated by the Kaplan-Meier curve (Ranstam and Cook, 2017) and log-rank test (Kleinbaum, 1998). The ROC curve (Kamarudin et al., 2017) and the AUC, C-index, Calibration analysis, and Decision Curve Analysis (Vickers and Elkin, 2006) were performed by “timeROC,” “rmda,” and “survcomp” packages to evaluate the risk model and the nomogram. Similarly, we evaluated the prediction efficiencies of the risk score system in the testing sets and GEO validation sets too.

### The Cutoff Value of the Km Curve

To better evaluate the validation model and the whole cohort model, we obtained a relatively fixed cutoff value by “Surv_cutpoint” function through the training cohort. This can ensure that the corresponding cutoff value will not be biased after different groups, and the verification of the model will be relatively more accurate. This cutoff value is only the best cutoff value obtained by the training group. This cutoff value will vary with the sample changes. Each cohort was divided into high-risk groups and low-risk groups according to their respective cutoff value.

### Gene Co-expression Network and Gene Functional Enrichment Analysis

Genes which co-expressed with the 12 risk-related genes were selected by the Pearson correlation method in TCGA-COAD high-risk group, low-risk group, and normal samples, and *p* < 0.05 were considered as significant. The co-expressed genes with Pearson correlation coefficient |R| > 0.6 were converted into a Topological Overlap Matrix (TOM) by “plotNetworkHeatmap” in the “WGCNA” package (Friedman et al., 2010), and the co-expressed genes with Pearson correlation coefficient |R| > 0.7 were converted into gene co-expression network by “network_plot” in the “correlate” package.

Gene ontology (GO) term analysis, Kyoto Encyclopedia of Genes and Genomes (KEGG, http://www.genome.jp/kegg/) pathway enrichment analyses were then performed with the “clusterprofiler” package to investigate the biological functions and pathway of the genes list used in the TOM heatmap. Gene set enrichment analysis (GSEA, https://software.broadinstitute.org/gsea/index.jsp) was used to analyze signaling pathway enrichment in high‐ and low-risk groups. The result of the enrichment analysis of biological functions and pathways were displayed by visual graphics. The top 10 most significant results of BP (biological process), CC (cellular components), MF (molecular function), and KEGG were selected, respectively. The GSEA analysis was performed with the following settings: FDR < 0.25, NOM value of *p* < 0.05, and |NES| > 1.

## Result

### Characteristics of COAD Patients in the TCGA Dataset and GEO Dataset

We enrolled 439 patients with follow-up time >30 days in total as the discovery set for construction and validation of the model. 263 and 176 patients were separated by random into two groups: the training group and the testing group. The patient characteristics of the training set and test set were in balance (*p* > 0.1). The average age in years was 66.8 ± 12.2, and 119 females (45.2%) in the training set; while the average age in years was 65.4 ± 13.4, 85 females (48.2%) in the testing set (Table 1).

**Table 1:** TGCA patient characteristics.

Variable		Number			
		Total set	Training set	Testing set	*p* value
Case		439	263	176	/
Gender	Female	204	119	85	0.396
	Male	235	144	91	
Survival status	Alive	346	210	136	0.811
	Dead	93	53	40	
Endpoint time		2.4 ± 2.0	2.5 ± 2.2	2.3 ± 1.8	0.943
Age		66.3 ± 12.7	66.8 ± 12.2	65.5 ± 13.4	0.649
M	M0	324	194	130	0.994
	M1	61	39	22	
	MX	49	27	22	
N	N0	258	149	109	0.574
	N1	103	65	38	
	N2	78	49	29	
T	T1	11	6	4	0.313
	T2	78	42	36	
	T3	299	174	125	
	T4	51	40	11	
	NA	11	6	5	
Stage	STAGE I	75	41	34	0.499
	STAGE II	167	100	67	
	STAGE III	125	77	48	
	STAGE IV	61	39	22	

NA, not reported.

Meanwhile, we downloaded four eligible datasets (GSE17538, GSE38832, GSE44861, and GSE44076) from GEO. However, two datasets (GSE44861 and GSE44076) were discarded for containing only 8 of 12 related genes we screened out, and the other two datasets (GSE17538 and GSE38832) containing 11 of 12 related genes are kept as validation datasets. Using the same exclusion criteria of the training group, 232 colon cancer patients out of a total of 238 samples were selected from GSE17583 datasets [average age in years was 64.7 ± 13.4, 110 females (47.4%)]. GSE38832 contains 122 colon cancer patients with disease-free survival and disease-specific survival information, but not overall survival information. Characteristics of patients in the training set, testing set of TCGA, GSE17583, and GSE38832 are summarized in Table 2.

**Table 2:** GEO patient characteristics.

GSE17583			GSE38832		
Case	232		Case	122	
Gender	Female	110	dfs time (year)	3.84 ± 2.77	
	Male	122			
Survival status	Alive	139	dfs status	no recurrence	83
				recurrence	9
				NA	30
	Dead	93			
Endpoint time (year)		3.95 ± 2.56	dss status	no death	94
Age		64.73 ± 13.43		death from cancer	28
Ajcc stage	1	28	Ajcc atage	1	18
	2	72		2	35
	3	76		3	39
	4	56		4	30
Tumor differentiation	WD	17	/		
	MD	235			
	PD	30			

NA, not reported; WD, well differentiated; MD, moderately differentiated; PD, poorly differentiated.

### Selection of DNA Damage and DNA Repair Related Genes and Construction of the Signature

**Figure 1:**
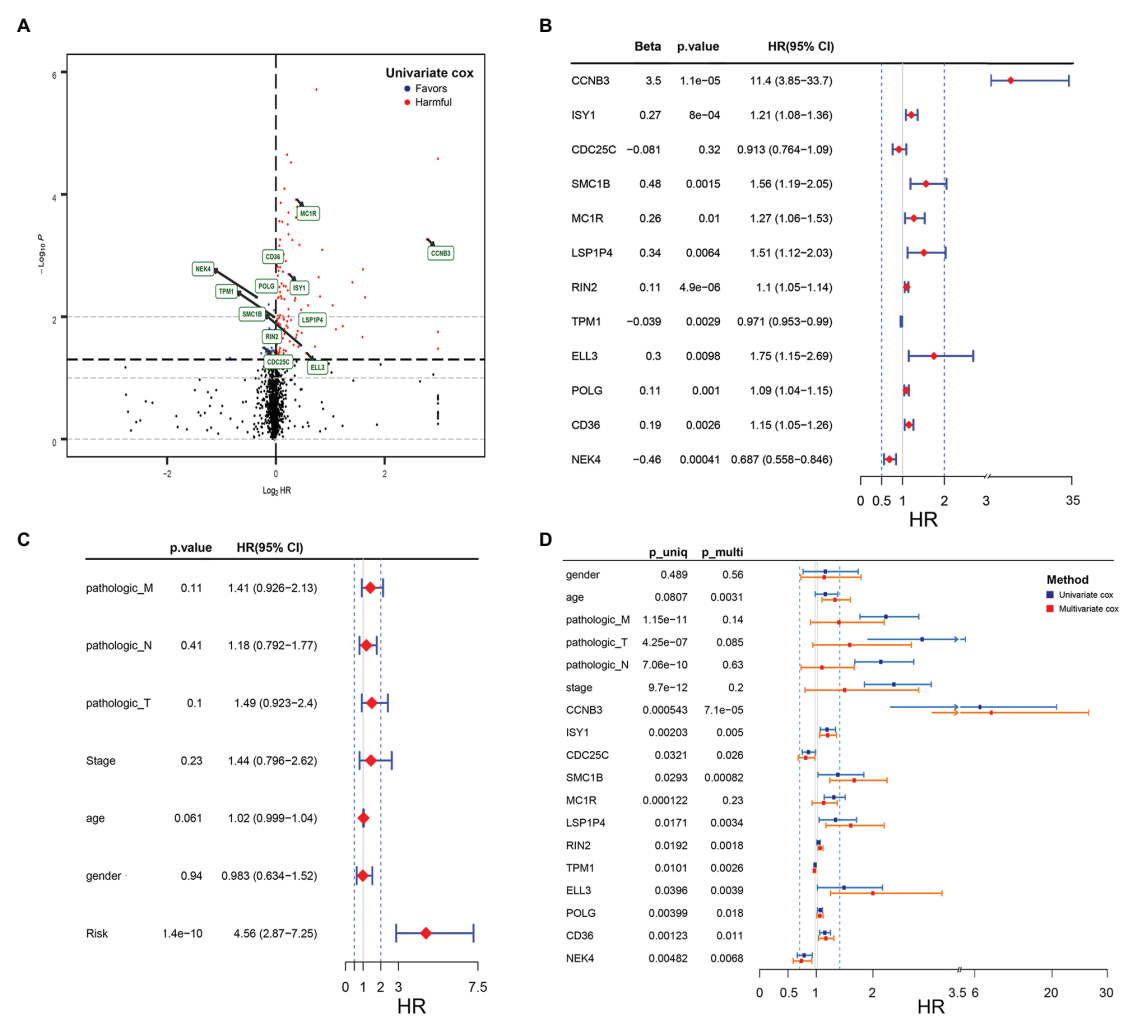
Volcano plot of DNA damage and repair related genes and forest plot of the multivariate Cox regression analysis in TCGA cohorts. **(A)** Volcano plot of DNA damage and repair related genes: blue indicates protective genes, red indicates harmful genes and black indicates no significance genes. **(B)** Forrest plot of the multivariate Cox regression analysis OS of 12 genes. **(C)** Forrest plot of the multivariate Cox regression analysis OS of clinical factors and risk score. **(D)** Forrest plot of the multivariate Cox regression analysis OS of clinical factors and 12 genes. Beta values represent the coefficient index *β* for each gene and clinical factors.

In the training set, univariate Cox regression analysis was performed for all the DNA damage and repair related genes selected from GSEA. As shown in Figure 1A, 27 DNA damage and repair related genes play a favorable role for COAD patients’ survival (blue, Hazard Ratio (HR) < 1, *p* < 0.05), and 133 genes were in risk roles (red, HR > 1, *p* < 0.05), while 1,385 gene showed no significance. Twelve genes were selected by stepwise multivariate regression analysis as reliable predictors, including CCNB3, ISY1, CDC25C, SMC1B, MC1R, LSP1P4, RIN4, TPM1, ELL3, POLG, CD36, and NEK4 (Figure 1B). All the above genes except CDC25C show an independent prognostic manner (*p* < 0.05). Among them, CCNB3, ISY1, SMC1B, MC1R, LSP1P4, RIN2, ELL3, POLG, and CD36 may be considered as oncogenes, whereas CDC25C, TPM1, and NEK4 may be tumor suppressor genes. The coefficients of these DNAs indicated their impact on survival prediction. Subsequently, the risk score system for TCGA-CAOD samples based on the expression level and the corresponding beta value of each gene was constructed by the following formula:

RS = (3.5) × ExpCCNB3 + (0.27) × ExpISY1 + (−0.081) × ExpCDC25C + (0.48) × ExpSMC1B + (0.26) × ExpMC1R + (0.34) × ExpLSP1P4 + (0.11) × ExpRIN4 + (−0.039) × ExpTPM1 + (0.3) × ExpELL3 + (0.11) × ExpPOLG + (0.19) × ExpCD36 + (−0.46) × ExpNEK4.

**Figure 2:**
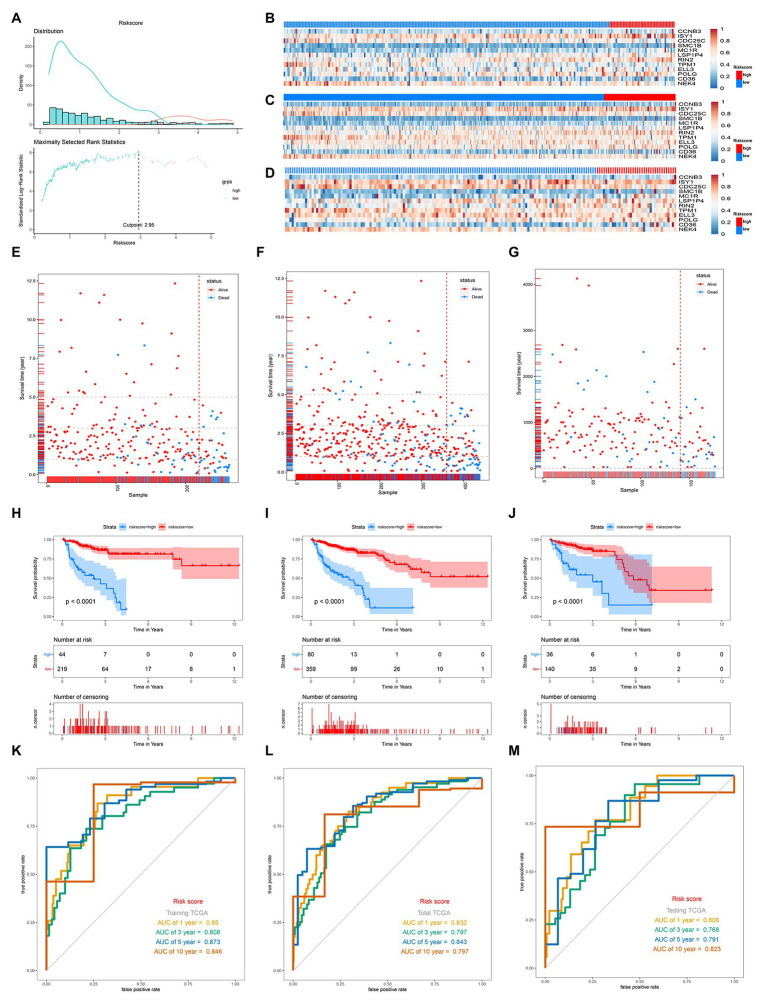
Distribution of risk score, Gene expression heatmaps, Kaplan-Meieranalysis and ROC analysis of 12-gene signature in the training TGCA set, total TCGA set, and testing set. **(A)** Distribution of risk score and the cutoff point. **(B–D)** Gene expression heatmaps in the training TGCA cohort **(B)**, total TCGA cohort **(C)**, and testing TCGA (**D**; The blue color is the low-risk group and the red color is the high-risk group). **(E,F)** Correlation between the prognostic signature and the OS of patients in the training TGCA cohort **(E)**, total TCGA cohort **(F)**, and testing TCGA **(G)**. **(H–J)** Kaplan-Meier survival analysis of the low‐ and high-risk group patients in the training TGCA cohort **(H)**, total TCGA cohort **(I)**, and testing TCGA **(J)**. **(K–M)** ROC curve analysis according to the 1, 3, 5, 10-year survival of the area under the AUC value in the training TGCA cohort **(K)**, total TCGA cohort **(L)**, and testing TCGA **(M)**.

According to the optimal cutoff value of 2.95 simulated by “Surv_cutpoint” function in “survminer” package, the TCGA-COAD patients were classified into high‐ and low-risk sets (Figure 2A). The patients’ status, survival time, and DNA expression levels of the test TCGA set, total TCGA set, and training TCGA set are shown in Figures 2B–G.

**Figure 3:**
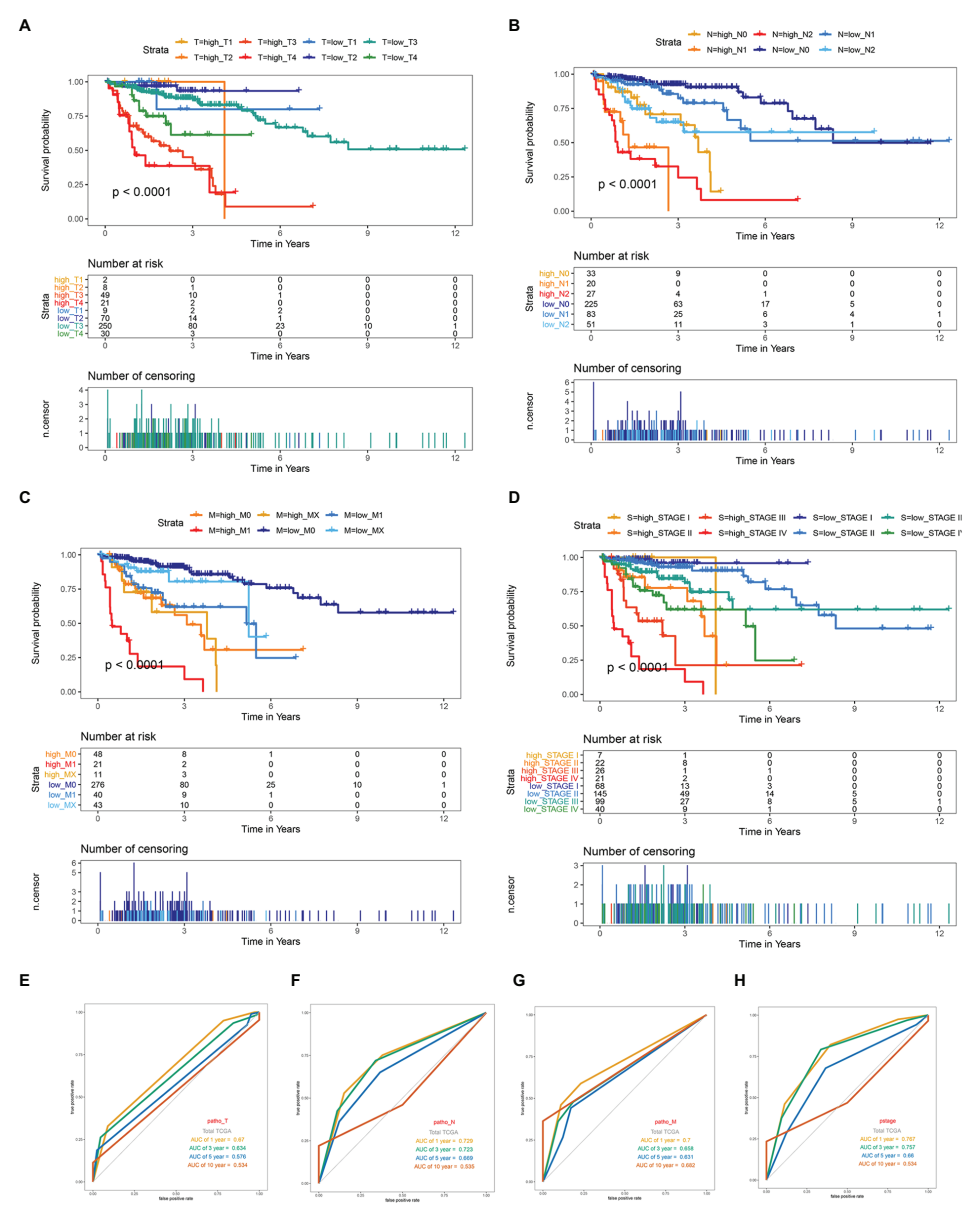
Kaplan-Meier survival for OS in high-risk and low-risk group of different subgroup and ROC curve analysis of T, N, M and stage in the total TCGA cohort. **(A)** In subgroups stratified by T1, T2, T3, and T4. **(B)** In subgroups stratified by N0, N1, and N2. **(C)** In subgroups stratified by M0, M1, and MX. **(D)** In subgroups stratified by stage I, stage II, stage III, and stage IV. **(E–H)** ROC curve analysis of T, N, M and stage according to the 1, 3, 5, and 10-year survival of the area under the AUC value in the total TCGA cohort.

The survival analysis presented that the OS of the low-risk set was better than that of the high-risk set in the training set of TCGA (hazard ratio, HR = 0.16, 95% confidence interval, 95% CI (0.1–0.24; Figure 2H). The results were consistent in the TCGA total set (HR = 0.138, 95% CI (0.079–0.24); *p* < 0.001; Figure 2I) and testing set (HR = 0.234, 95% CI (0.12–0.44); *p* < 0.001; Figure 2J). The 5-year survival rate for high and low risk is 11 and 79%, respectively, (Figure 2I). The area under the ROC curve (AUC) for 1-, 3-, 5-, and 10-year OS were all above 0.8 in the TCGA training set (Figure 2K), and in the TGCA total set (Figure 2L) and TCGA testing set (Figure 2M), they were all above 0.75. Meanwhile, we investigated the relationship between risk score and clinicopathologic features including T, N, M, and stage in the TCGA total cohort. As shown in Figures 3A–D, respectively comparing the clinical data of patients of the same T, N, M, and stage in the high-risk and low-risk groups, the prognosis of patients was significantly different. Under the same T, N, M, or stage, the survival time of patients in the low-risk group was longer than that of the high-risk group.

### Validation of the Genes Signature in GEO Dataset

**Figure 4:**
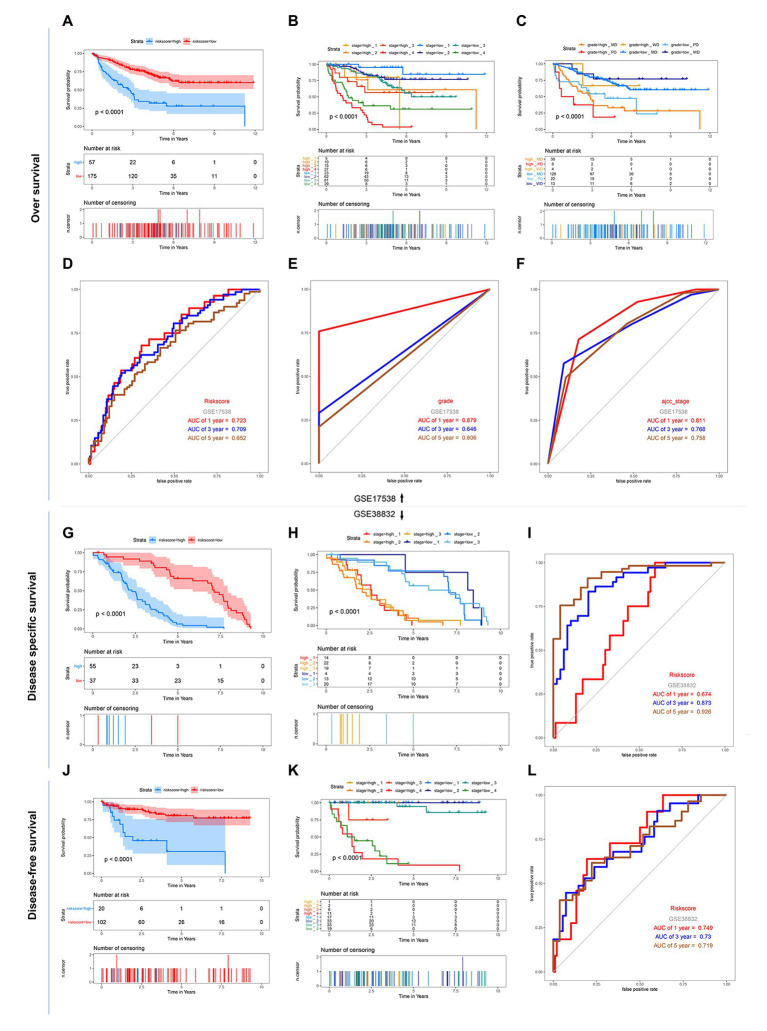
Kaplan-Meier survival and ROC curves of the 12-DNA signature, grade and stage in the two GEO sets. **(A)** Correlation between the 12-DNA signature and the overall survival of patients in the GSE 17538 set. (**B,C**) Kaplan-Meier survival for OS in high-risk and low-risk group of different subgroup in the GSE 17538 set: in subgroups stratified by stage I, stage II, stage III, and stage IV, in subgroups stratified by grade MD, grade PD, and grade WD. **(D–F)** ROC curve analysis of risk score, stage and grade according to the 1, 3, 5, and 10-year survival of the area under the AUC value in the GSE 17538 set. **(G)** Correlation between the 12-DNA signature and the disease specific survival of patients in the GSE 38832 set. **(H)** Kaplan-Meier survival for disease specific survival in stage 1, 2, and 3 subgroups of high-risk and low-risk group in the GSE 38832 set. **(I)** ROC curve analysis of risk score according to the 1, 3, and 5-year disease specific survival of the area under the AUC value in the GSE 38832 set. **(J)** Correlation between the 12-DNA signature and the disease-free survival of patients in the GSE 38832 set. **(K)** Kaplan-Meier survival for disease-free survival in stage 1, 2, 3, and 4 subgroups of high-risk and low-risk group in the GSE 38832 set. **(L)** ROC curve analysis of risk score according to the 1, 3, and 5-year disease-free survival of the area under the AUC value in the GSE 38832 set.

GSE17538 and GSE38832 datasets both based on the platform of Affymetrix-GPL570 included the 11 above risk-related genes except LSP1P4 were used for the following analysis. The results showed that though the new gene signature missing a significant gene, the 11-gene based signature still had a significant performance for OS, DFS, and DSS prediction in the two GEO validation datasets (Figures 4A,D,G,I,J,L). The relationship between risk score of “ajcc_stage” and tumor differentiated grade was also investigated in the two sets, which showed that in the same stage or differentiated level, the survival time of patients in the low-risk group was apparently longer than that of the high-risk group (Figures 4B,C,H,K), similar to the results in the training set. Together, we considered that the 11-gene signature had a prominent prognostic ability not only for OS prediction but also DFS and DSS prediction.

### Comparison of the Prognostic Performance of Genes Signature With Clinical Predictive Factors

Given the fact that T, N, M, and stage have been thought to be predictive factors of the prognosis of COAD in the past, we managed to compare the prognostic performance of these clinicopathologic features with our 12-gene signature. Survival analysis of the above clinical indicators was completed, respectively, in the high-risk and low-risk groups (Figures 2L, 3E-H). The survival analysis presented that these clinicopathologic features showed less satisfactory performance for OS prediction than that of 12-gene signature. The area under the ROC curve (AUC) for 1-, 3-, 5-, and 10-year OS of T (the size of the tumor) were 0.67, 0.634, 0.576, and 0.543 in the total TCGA set, comparing with the AUC of 12-gene signature in the total TCGA set (0.832, 0.797, 0.843, and 0.797). The results were consistent in the GEO colon cancer validation sets containing not only COAD patients (Figure 4). Combining the above results, a 12-gene signature can be used as a satisfactory indicator to predict the prognosis of COAD patients or the whole colon cancer types.

### Establishment and Validation of the Nomogram Survival Model

By the usage of multivariable Cox regression analyses, pathologic M, pathologic T, pathologic N, stage, age, gender, and risk score status were selected to assess the independent prognostic manner in the COAD samples. Based on the result shown in Figure 1C, the risk score can be used as an independent prognostic factor without being affected by clinicopathologic features. And the HR of the high-risk group is 4.56 (2.87–7.25) times danger than that of the low-risk group (Figure 1C) The result of the multivariable Cox regression analysis of 12 genes along with clinicopathologic features was revealed in Figure 1D, indicating that most of these genes except MC1R can also act as independent prognostic factors, and may have an excellent suggestive effect on predicting the survival of COAD patients. Among these genes, CCNB3, ELL3, LSP1P4, and SMC1B showed a significant harmful effect on COAD OS (HR > 1.5, *p* < 0.05).

**Figure 5:**
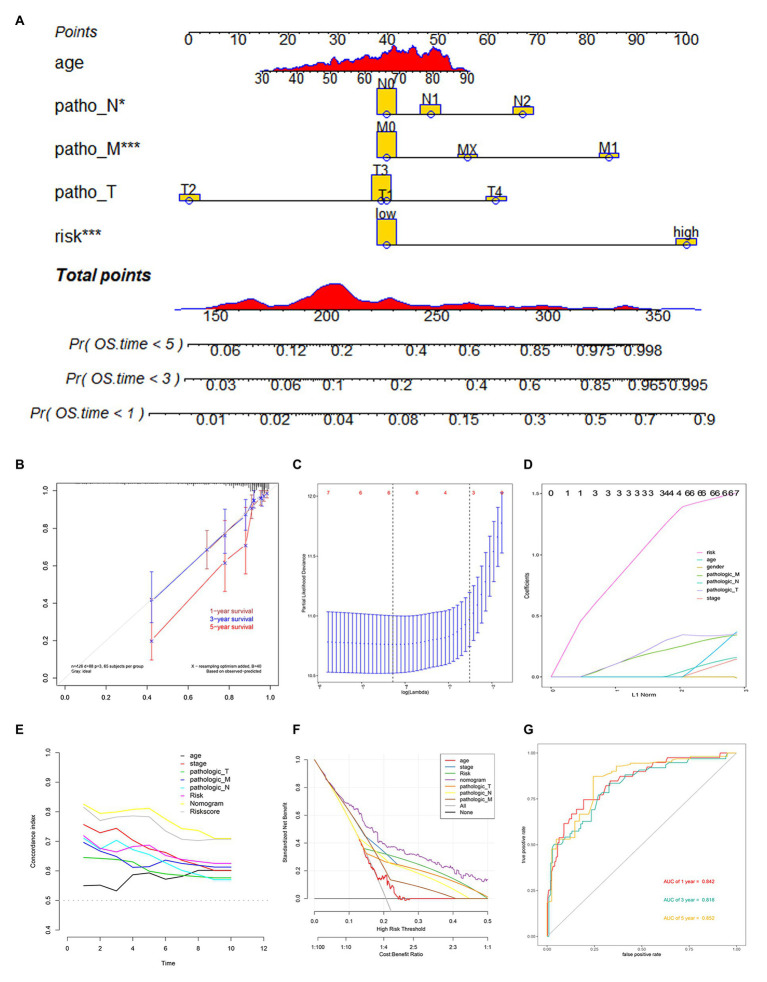
Nomogram construction based on 12-gene signature and prognostic value of 12 genes. **(A)** The nomogram for predicting the proportion of patients with 1-, 3-, or 5-year OS. **(B)** Calibration plots of the nomogram. **(C,D)** LASSO regression analysis used 10-fold cross-validation *via* the maximum criteria. **(E)** C-index of the nomogram **(F)** Decision curve analysis of nomogram predicting 1-, 3-, and 5-year OS of COAD comparing age, stage, the risk score, Pathologic T, Pathologic N, and Pathologic M. **(G)** Time-dependent ROC analysis of nomogram predicting 1-, 3-, and 5-year OS of COAD.

To establish a clinical method to predict the survival probability of COAD patients, we created a nomogram by LASSO regression analysis based on the TCGA cohort to estimate the probability of the 1-, 3-, and 5-year OS with T, N, M, age, gender, stage, and risk group status (Figure 5A). LASSO regression analysis established that the nomogram contained 5 prognostic factors including age, T, M, N, and risk (Figures 5C,D). The AUC of 1-, 3-, and 5-year OS predictions all above 0.8 (Figure 5G).

Calibration curves were used to evaluate the consistency between actual and predicted survival rates. As shown in Figure 5B, the accuracy of this model in predicting a 5-year survival rate is low, but in predicting a 1‐ and 3-year survival rate it is high, showing that the nomogram was best for predicting 1-, 3-year OS in COAD patients. The concordance index (C-index) was calculated to evaluate the model prognosis capability. The values of 0.5 and 1.0 represent a random probability and an excellent performance for predicting survival with the model. The C-index of the risk score and nomogram were all above 0.75 between the 1–5 years OS prediction, which was much better than any other independent predictor (Figure 5E). We used DCA analysis to confirm a range of threshold probabilities for a prediction mode, as shown in Figure 5F, the nomogram threshold probability based on 12-gene combinations was significantly better than the default strategies of treating all or none at a threshold probability more than 0.1, and the results come better than any other predictor used in this study.

### Function and Signaling Pathways Analysis of Genes in the Prognosis Module

**Figure 6:**
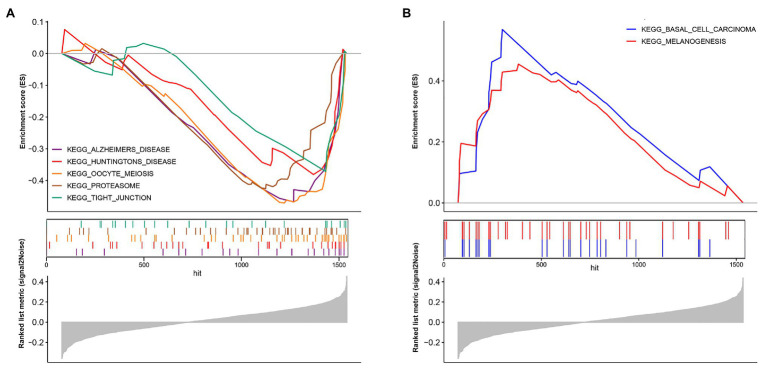
Biological pathways in two different risk groups by GSEA analysis. **(A)** Enriched pathways in the low-risk group. **(B)** Enriched pathways in the high-risk group.

The model constructed by 12 genes can effectively distinguish patients with different prognoses, which suggests that patients with different risk scores may be involved in different important pathways that cause differences in the final prognosis. Based on the above conjectures, we performed GSEA analysis in high‐ and low-risk patients, respectively, to confirm the significant pathways in each group. According to the enrichment results, two different groups have their characteristic pathways. Multiple pathways such as Alzheimers disease, Huntingtons disease, Oocye meiosis, Proteasome, and Tight junction are downregulated in patients with a low-risk score (Figure 6A). On the other hand, in the high-risk group, two pathways, including Basal cell carcinoma and Melanogenesis, were up-regulated (Figure 6B).

**Figure 7:**
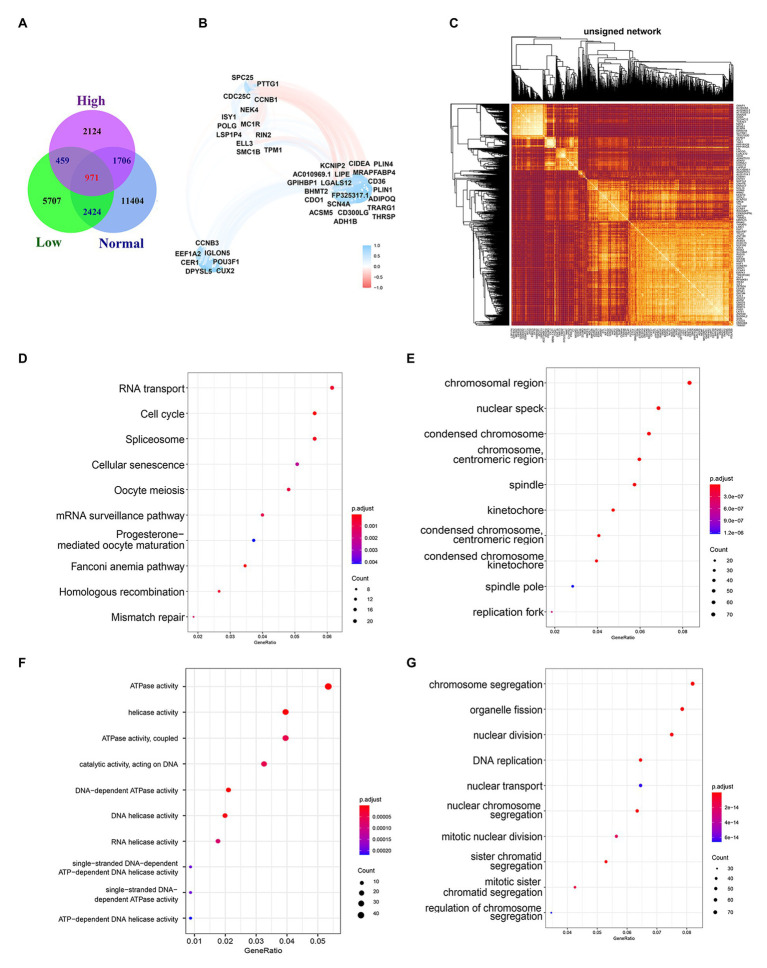
Biological functions and pathways of co-expressed genes. **(A)** Venn diagram of overlapping genes among Normal group, Low-risk group, and High-risk group. **(B)** Topological overlap heatmap of gene co-expression network. Dark colors mean high topological overlap, while Light colors mean low topological overlap. **(C)** Co-expressed genes selected by R2 > 0.7. **(D)** The top 10 most significant results of KEGG. **(E–G**) The GO enrichment analysis of co-expressed genes, including the CC **(E)**, the MF **(F)**, and the BP **(G)**.

Biological processes are often not the result of the action of a single gene but are often realized through the interaction between genes. Considering that gene expression varies in different individuals and different statuses, we searched for genes related to 12 genes in the normal group, low-risk group, and high-risk group and took the intersection of the three as the gene group of 12 genes co-expression. We used R = 0.6 and *p* < 0.01 as the cutoff value and the correlation with any one of the 12 genes met the condition that they were included in the statistics. Finally, 16,505, 9,561, and 5,260 (including 12 genes) were found in the normal group, low-risk group, and high-risk group, respectively (Figure 7A). The number of genes related to 12 genes is the largest in the normal group and the least in the high-risk group, which is related to tumor heterogeneity. The lowest number of genes in high-risk patients suggests more significant heterogeneity, which is consistent with the final poor prognosis. We used WGCNA to build the Topological Overlap Matrix (TOM), which proved that the selected gene group has a good correlation (Figure 7C). Next, we further screened the related genes with a cutoff value > 0.7, resulting in a total of 42 genes including the genes of the module. These genes are roughly classified into three clusters, most of the 12 genes (10/12) are located in the upper left corner, and there is a clear correlation between the other two clusters of genes, which further proves the relative independence of the genes of the module and the reliability of the co-expressed genes (Figure 7B).

GO enrichment analysis and KEGG pathway enrichment analysis are performed to investigate the biological functions and pathways of the Co-expressed genes. The results of KEGG enrichment analysis showed that the co-expressed genes were significantly enriched in important biological pathways, such as RNA transport, Cell cycle, Spliceosome, and so on (Figure 7D). The cellular components (CC) analysis indicated that proteins encoded by genes were mostly located in the chromosomal region, nuclear speck, condensed chromosome, chromosome centromeric region, and spindle (Figure 7E). Those molecular function (MF) were significantly associated with ATPase activity, helicase activity, ATPase activity coupled, catalytic activity acting on DNA, and so on (Figure 7F). For the biological process (BP), genes were mainly enriched in chromosomal segregation, organelle fission, nuclear division, DNA replication (Figure 7G).

## Discussion

COAD has one of the highest fatality rates of tumors in the digestive system. It is more common in men over the age of 40. However, early diagnosis of COAD was extremely difficult, and many patients have progressed to advanced cancer when they are diagnosed with COAD, leading to a bad prognosis. Early diagnosis and treatment of COAD can greatly improve the prognosis of COAD patients, which will not only reduce the economic burden of patients but also improve the quality of life. TNM staging is the one that is currently widely used, but this staging has certain drawbacks, and differences in treatment options may have caused unexpected differences in survival outcomes. For example, patients with stage IIIA disease receiving chemotherapy have better survival than those with stage IIB disease, where the survival difference is based on the benefit of chemotherapy or whether the stage IIA tumor itself is unknown according to aggressiveness (O’Connell et al., 2004). Meanwhile, with the intensive research on the molecular mechanism of tumors, the advantage of prognosis prediction based on gene-level is gradually exhibited. For example, colorectal cancers (CRCs) are classified into MMR and MMR-d based on whether they have normal DNA mismatch repair (MMR) function, a phenotype that is also an important prognostic indicator. It has been controversial whether the MMR-d/MSI-H phenotype benefits from 5-fluorouracil – based chemotherapy (Stadler, 2015). Therefore, the discovery, identification, and evaluation of new biomarkers are greatly important for COAD patients.

By consulting the previous literature, DNA damage and repair have been proved to be related to the proliferation and metastasis of CRC, but there is no research to clarify its direct relationship with the prognosis or consider DNA damage and repair related genes as prognosis predictors, which serves as the breakthrough point of our research. DNA is constantly on the exposure to endogenous and exogenous sources of damage, destroying genomic integrity (Hoeijmakers, 2009). Unable to repair DNA damage in a precise and well-timed way will lead to various genomic aberrations, including point mutations, chromosomal translocations, and the acquisition or loss of chromosomes. The accumulation of these aberrations will further cause changes in the cells, thus driving the tumorigenesis (Burrell et al., 2013; Khanna, 2015; Jeggo et al., 2016). The contrasting activity of multiple DNA repair pathways plays a key role in interrupting this accumulation and maintaining genomic integrity (Mouw et al., 2017). DNA repair and damage have been described as being related to the occurrence and development of various cancers, such as breast cancer and ovarian cancer. So, we legitimately speculated that DNA damage and repair were closely related to the development of CRC. We used DNA damage and repair related gene sets collected from GSEA gene sets and TCGA-COAD cohort to assess their diagnostic value.

The fast development of sequencing technology produces massive data, which facilitates tumor biomarker identification and a lot of resources have been invested in corresponding research. For example, Yang et al. (2019) construct a prognosis model based on the methylation profiles of 18 CpG that can help to identify new biomarkers, precise drug targets, and molecular subtype classification of COAD patients. Ma et al. (2019) constructed a 10 differentially expressed microRNA prediction model that has high accuracy for OS. In this study, we constructed 12 DNA damage and repair related genes which showed a significant performance for OS prediction in the TCGA cohort and two GSE validation cohort. ROC, DCA, KM, and C-index all proved the 12-gene signature could be an excellent predictor for OS prediction. Meanwhile, we built a nomogram survival model to predict 1/3/5 years survival rate by combining Pathologic M, pathologic T, pathologic N, age, and stage.

There is a point worth making, all the samples included in the TCGA database were COAD, however, the samples in the GEO database include all types of colon cancer and the model constructed by TCGA has 12 genes, while the GEO database only contains 11 of them, which leads to the result that the model has an ideal prediction effect in the train and test groups of TCGA, while the validation effect in GEO is not as good as that in TCGA. We also note that one of the GEO databases only have DFS and DSS information to illustrate our model established by COAD samples. Relapse or tumor-induced death also has a good predictive function, but there is no other corresponding data to verify. In our research, we also refer to a novel web analysis tool suite, TSUNAMI, which can be used for data download, preprocessing and enrichment analysis (Huang et al., 2019).

After reviewing the existing literature, we found that the 12 genes are more or less related to tumors. The cyclin B1-Cdk1 complex is a key regulator of a large number of phosphorylated proteins mitotic entry. Regulation of the mitotic events is linked to activity control of the cyclin B1-Cdk1 complex to make cells enter mitosis, arrest at G2-phase, or skip mitosis (Nakayama and Yamaguchi, 2013). Base excision DNA repair (BER) is the most vital pathway to remove oxidized or mono-alkylated DNA, and APE1 is an important multifunctional enzyme in BER. Oxidative damage induces ISY1 expression. This gene promotes the 5'-3' endonuclease activity of APE1, thereby enhancing the repairability of DNA damage in the cell genome (Jaiswal et al., 2020). Cell Division Cycle 25C (CDC25C) plays an important role in the regulation of G2/M processes and mediates DNA damage repair by checkpoint protein regulation in case of DNA damage. The abnormal expression of cdc25c is related to tumorigenesis and development, and it is a promising therapeutic target (Liu et al., 2020). A large number of mitochondrial DNA (mtDNA) deletion is related to many human diseases and aging. DSB (Double-Strand Breaks) is one of the causes of mtDNA deletion. The exonuclease function of POLG can quickly degrade mtDNA fragments, which minimizes the effect of DSB on mtDNA deletion. The abnormality of POLG will eventually increase the deletion of mtDNA, which has been confirmed in mutant and aging individuals (Nissanka et al., 2018). SMC1B exists in mammalian somatic cells and is related to mitotic cohesion proteins, which help to maintain genome stability and the normal process of gene transcription (Mannini et al., 2015). SMC1B is found to be mutated in UBC and plays an important role in it (van der Lelij et al., 2017). Ras and Rab interactor 2 (RIN2) can associate with GTP-bound Rab5 and take part in early endocytosis (Syx et al., 2010). This gene and SLC22A18, PIGR, and GJA12 can effectively divide Barrett’s Esophagus into three groups with different risks and can detect dysplasia/early-stage neoplasia (Alvi et al., 2013).TPM1, as a tumor suppressor gene, was found to be significantly downregulated in colorectal cancer, mainly because of epigenetic and genetic events, which are closely related to the occurrence of colorectal cancer (Mlakar et al., 2009). ELL3 is encoded by an androgen-response gene in the prostate, and it is homologous with ELL and ELL2 (Miller et al., 2000). It was found that the lack of ELL significantly hindered the transcription resumption of RNA Pol II (RNA polymerase II) after DNA repair and increased the RNA Pol II retention to the chromatin, which proved to be an important member of RNA Pol II restart and participated in the transcription recovery after DNA repair (Mourgues et al., 2013). Through bioinformatics methods, CD36 was found to be associated with lipid metabolism and immune response (Hao et al., 2019), and its high expression was associated with poor prognosis of COAD, and it was found that CD36 was the target of quercetin on COAD (Pang et al., 2019). MC1R is a G-protein-coupled receptor, can cause increased pigmentation, G 1-like cell cycle arrest induced by ultraviolet B, and control senescence and melanoma *in vivo* and *in vitro*, which plays a central role in the prevention of melanoma (Chen et al., 2017). The expression of CCNB3 is usually limited to the testis and encodes a protein with premeiotic function, CyclinB3. CCNB3 can form a fusion gene with BCOR, BCOR-CCNB3, which defines a new subtype of bone sarcoma (Astolfi et al., 2019). NEK4 encodes NIMA-related kinase 4. Inhibition of NEK4 can lead to decreased response to DNA damage and damage the anti-tumor activity of p53. NEK4 is expressed in different stages of CRC, with the highest expression in stage I patients and the lowest expression in stage IV patients. It indicates that a low level of NEK4 is an adverse prognostic factor in CRC patients (Huo et al., 2017). Collectively, we suggested our 12-DNA signature and nomogram could be practical and reliable prognostic tools for COAD. In terms of COAD’s overall survival prediction, they can provide higher clinical value than traditional prediction systems and utilize treatment decisions.

Through the gene functional enrichment analysis of 12 genes and their co-expressed genes, we can find that 12 genes are involved in the occurrence and development of COAD by participating in a variety of important biological pathways, meanwhile, through GSEA analysis, we found that there were different pathways in the high‐ and low-risk group. For example, in the low-risk patient group, it is mainly concentrated in Alzheimers disease, Huntingtons disease, Oocye meiosis, Proteasome, and Tight junction, in which Tight junction is closely related to intestinal inflammation and the occurrence of intestinal tumor (Sharma et al., 2018). The proteasome pathway is widely studied, thanks to the proteasome’s ability to control cellular protein quality by degrading misfolded or damaged proteins, which is also key to tumor cell survival (Konstantinopoulos and Papavassiliou, 2006). UPP (The ubiquitin-proteasome pathway) abnormalities play an important role in the occurrence and development of colon cancer. For example, APC (Adenomatous Polyposis Coli) gene mutations in patients with familial adenomatous polyposis syndrome can promote the occurrence of final colon cancer (Konstantinopoulos and Papavassiliou, 2006).

Although the 12-gene signature and nomogram showed excellent performance in the training set and test sets, it had the following defects. First, the gene signature was built with 12-genes but validated by 11-genes in the GEO cohort for the GEO database only contains 11 of them. A relative NRI analysis showed that the 12-gene model performed better than the latter model (Supplementary Figures 1A–C). The NRI > 0 for the difference between the two model predictions of the 1, 3, and 5 year survival. This means that the 12-gene model has improved predictive ability compared to the 11-gene model. Meanwhile, though missing a significant gene, the predictive ability for OS, DFS, and DSS of the risk model was significant in the two GEO validation datasets, as we have shown in the results. Second, although the 12-gene signature performed well in predicting the survival of COAD patients, it lacked the verification of large-scale prospective trials. Third, all the samples included in the TCGA database were COAD, while the samples in the GEO database include all types of colon cancer. The TCGA data is gene sequencing data while the GEO data is gene chip data, these differences may mean that the results to come from the validation data may not fully reflect the real prognostic effect of these genes on COAD. And finally, the associated mechanisms had not been validated in COAD cells. Based on this, our follow-up research will focus on verifying the conclusions of this study in terms of clinical application and molecular mechanisms.

In conclusion, we introduced a 12-gene signature which might be an independent prognostic factor in COAD and a novel nomogram that could predict the survival of COAD patients.

## Data Availability Statement

The datasets presented in this study can be found in online repositories. The names of the repository/repositories and accession number(s) can be found in the article/Supplementary Material.

## Author Contributions

X-qW, S-wX, WW, S-zP, X-bZ, and YW participated in the design of the study and performed the statistical analysis. X-lM, W-dW, L-pY, and S-wL drafted the manuscript. All authors contributed to the article and approved the submitted version.

### Conflict of Interest

The authors declare that the research was conducted in the absence of any commercial or financial relationships that could be construed as a potential conflict of interest.

## Footnotes

^1^https://www.gsea-msigdb.org/gsea/index.jsp

^2^https://cancergenome.nih.gov/

^3^https://www.ncbi.nlm.nih.gov/geo/

## Supplementary Material

Supplementary Material The process and results of stepwise.

Supplementary Figure 1 The NRI and IDI analysis results for the 1year, 3 year, and 5 years survival prediction using a 12 gene model and 11 gene model. (A) 1 year, (B) 3 year, and (C) 5 year.

Supplementary Table 1 The information of 1545 genes related to DNA damage and repair.
