# Supplementary material for: Identification and Validation of a Novel DNA Damage and DNA Repair Related Genes Based Signature for Colon Cancer Prognosis
Source: Front Genet. 2021 Feb 24;12:635863. doi: 10.3389/fgene.2021.635863 (PMC7943631; doi:10.3389/fgene.2021.635863)
Supplement: Supplementary Material — The process and results of stepwise. [file Data_Sheet_1.docx]

My.stepwise.coxph

After the univariate Cox regression to screen out meaningful genes (p<0.05). Corresponding to the filtered variables, we used the My.stepwise.coxph function in the My. stepwise package to build the model. In order to get each factor to be an independent prognostic factor, we set SLE (significance level for entry) and SLS (significance level for stay) to 0.05, which is smaller than the default value of 0.15.

Functions:

My.stepwise.coxph (Time = NULL, T1 = NULL, T2 = NULL, Status = NULL, variable.list, in.variable = "NULL", data, sle = 0.05, sls = 0.05, vif.threshold = 999).

The Stepwise multivariate cox results and the finial model are as follows

**## Initial Model:**

CCNB3 2.1049 8.2060 0.5373 3.918 8.94e-05 ***

---

Signif. codes: 0 ‘***’ 0.001 ‘**’ 0.01 ‘*’ 0.05 ‘.’ 0.1 ‘ ’ 1

exp(coef) exp(-coef) lower .95 upper .95

CCNB3 8.206 0.1219 2.863 23.52

Concordance= 0.557 (se = 0.034 )

Likelihood ratio test= 9.03 on 1 df, p=0.003

Wald test = 15.35 on 1 df, p=9e-05

Score (logrank) test = 15.29 on 1 df, p=9e-05

# --------------------------------------------------------------------------------------------------

**### iter num = 1, Forward Selection by LR Test: + `NEK4`**

coef exp(coef) se(coef) z Pr(>|z|)

CCNB3 2.58096 13.20985 0.52596 4.907 9.24e-07 ***

NEK4 -0.30746 0.73531 0.09102 -3.378 0.00073 ***

---

Signif. codes: 0 ‘***’ 0.001 ‘**’ 0.01 ‘*’ 0.05 ‘.’ 0.1 ‘ ’ 1

exp(coef) exp(-coef) lower .95 upper .95

CCNB3 13.2099 0.0757 4.7119 37.0340

NEK4 0.7353 1.3600 0.6152 0.8789

Concordance= 0.662 (se = 0.029 )

Likelihood ratio test= 21.04 on 2 df, p=3e-05

Wald test = 29.38 on 2 df, p=4e-07

Score (logrank) test = 29.84 on 2 df, p=3e-07

--------------- Variance Inflating Factor (VIF) ---------------

Multicollinearity Problem: Variance Inflating Factor (VIF) is bigger than 10 (Continuous Variable) or is bigger than 2.5 (Categorical Variable)

CCNB3 NEK4

1.071633 1.071633

# --------------------------------------------------------------------------------------------------

**### iter num = 2, Forward Selection by LR Test: + `ISY1`**

coef exp(coef) se(coef) z Pr(>|z|)

CCNB3 2.57988 13.19561 0.51964 4.965 6.88e-07 ***

NEK4 -0.35988 0.69776 0.09729 -3.699 0.000217 ***

ISY1 0.20091 1.22252 0.05764 3.486 0.000491 ***

---

Signif. codes: 0 ‘***’ 0.001 ‘**’ 0.01 ‘*’ 0.05 ‘.’ 0.1 ‘ ’ 1

exp(coef) exp(-coef) lower .95 upper .95

CCNB3 13.1956 0.07578 4.7655 36.5382

NEK4 0.6978 1.43316 0.5766 0.8443

ISY1 1.2225 0.81798 1.0919 1.3687

Concordance= 0.676 (se = 0.029 )

Likelihood ratio test= 32.71 on 3 df, p=4e-07

Wald test = 42.53 on 3 df, p=3e-09

Score (logrank) test = 47.24 on 3 df, p=3e-10

--------------- Variance Inflating Factor (VIF) ---------------

Multicollinearity Problem: Variance Inflating Factor (VIF) is bigger than 10 (Continuous Variable) or is bigger than 2.5 (Categorical Variable)

CCNB3 NEK4 ISY1

1.100329 1.101745 1.044870

# --------------------------------------------------------------------------------------------------

**### iter num = 3, Forward Selection by LR Test: + `RIN2`**

coef exp(coef) se(coef) z Pr(>|z|)

CCNB3 2.77739 16.07696 0.52435 5.297 1.18e-07 ***

NEK4 -0.42579 0.65325 0.09919 -4.293 1.77e-05 ***

ISY1 0.20980 1.23343 0.05770 3.636 0.000277 ***

RIN2 0.06277 1.06478 0.01737 3.613 0.000303 ***

---

Signif. codes: 0 ‘***’ 0.001 ‘**’ 0.01 ‘*’ 0.05 ‘.’ 0.1 ‘ ’ 1

exp(coef) exp(-coef) lower .95 upper .95

CCNB3 16.0770 0.0622 5.7528 44.9295

NEK4 0.6533 1.5308 0.5378 0.7934

ISY1 1.2334 0.8107 1.1015 1.3811

RIN2 1.0648 0.9392 1.0291 1.1017

Concordance= 0.685 (se = 0.029 )

Likelihood ratio test= 44.48 on 4 df, p=5e-09

Wald test = 54.33 on 4 df, p=4e-11

Score (logrank) test = 58.48 on 4 df, p=6e-12

--------------- Variance Inflating Factor (VIF) ---------------

Multicollinearity Problem: Variance Inflating Factor (VIF) is bigger than 10 (Continuous Variable) or is bigger than 2.5 (Categorical Variable)

CCNB3 NEK4 ISY1 RIN2

1.102072 1.124490 1.052350 1.026844

# --------------------------------------------------------------------------------------------------

**### iter num = 4, Forward Selection by LR Test: + `POLG`**

**coef exp(coef) se(coef) z Pr(>|z|)**

CCNB3 2.61379 13.65065 0.53441 4.891 1.00e-06 ***

NEK4 -0.47712 0.62057 0.09872 -4.833 1.34e-06 ***

ISY1 0.23489 1.26477 0.05798 4.051 5.09e-05 ***

RIN2 0.07821 1.08135 0.01814 4.312 1.62e-05 ***

POLG 0.09476 1.09939 0.02357 4.020 5.83e-05 ***

---

Signif. codes: 0 ‘***’ 0.001 ‘**’ 0.01 ‘*’ 0.05 ‘.’ 0.1 ‘ ’ 1

exp(coef) exp(-coef) lower .95 upper .95

CCNB3 13.6506 0.07326 4.7892 38.908

NEK4 0.6206 1.61143 0.5114 0.753

ISY1 1.2648 0.79066 1.1289 1.417

RIN2 1.0814 0.92477 1.0436 1.120

POLG 1.0994 0.90959 1.0498 1.151

Concordance= 0.711 (se = 0.029 )

Likelihood ratio test= 58.45 on 5 df, p=3e-11

Wald test = 67.36 on 5 df, p=4e-13

Score (logrank) test = 68.96 on 5 df, p=2e-13

--------------- Variance Inflating Factor (VIF) ---------------

Multicollinearity Problem: Variance Inflating Factor (VIF) is bigger than 10 (Continuous Variable) or is bigger than 2.5 (Categorical Variable)

CCNB3 NEK4 ISY1 RIN2 POLG

1.084982 1.145386 1.084322 1.086162 1.144302

# --------------------------------------------------------------------------------------------------

**### iter num = 5, Forward Selection by LR Test: + `TPM1`**

CCNB3 2.582907 13.235560 0.534677 4.831 1.36e-06 ***

NEK4 -0.449700 0.637820 0.098448 -4.568 4.93e-06 ***

ISY1 0.238975 1.269947 0.058339 4.096 4.20e-05 ***

RIN2 0.090242 1.094439 0.018331 4.923 8.53e-07 ***

POLG 0.101419 1.106740 0.023984 4.229 2.35e-05 ***

TPM1 -0.022838 0.977421 0.008946 -2.553 0.0107 *

---

Signif. codes: 0 ‘***’ 0.001 ‘**’ 0.01 ‘*’ 0.05 ‘.’ 0.1 ‘ ’ 1

exp(coef) exp(-coef) lower .95 upper .95

CCNB3 13.2356 0.07555 4.6411 37.7451

NEK4 0.6378 1.56784 0.5259 0.7736

ISY1 1.2699 0.78743 1.1327 1.4238

RIN2 1.0944 0.91371 1.0558 1.1345

POLG 1.1067 0.90355 1.0559 1.1600

TPM1 0.9774 1.02310 0.9604 0.9947

Concordance= 0.726 (se = 0.027 )

Likelihood ratio test= 66.14 on 6 df, p=3e-12

Wald test = 72.47 on 6 df, p=1e-13

Score (logrank) test = 75.67 on 6 df, p=3e-14

--------------- Variance Inflating Factor (VIF) ---------------

Multicollinearity Problem: Variance Inflating Factor (VIF) is bigger than 10 (Continuous Variable) or is bigger than 2.5 (Categorical Variable)

CCNB3 NEK4 ISY1 RIN2 POLG TPM1

1.077802 1.163758 1.102224 1.094050 1.207719 1.101278

# --------------------------------------------------------------------------------------------------

**### iter num = 6, Forward Selection by LR Test: + `CD36`**

CCNB3 2.615042 13.667786 0.535500 4.883 1.04e-06 ***

NEK4 -0.435732 0.646791 0.099607 -4.375 1.22e-05 ***

ISY1 0.230959 1.259808 0.057786 3.997 6.42e-05 ***

RIN2 0.088018 1.092008 0.018870 4.664 3.09e-06 ***

POLG 0.102008 1.107393 0.024111 4.231 2.33e-05 ***

TPM1 -0.025750 0.974579 0.009357 -2.752 0.00592 **

CD36 0.128773 1.137432 0.042175 3.053 0.00226 **

---

Signif. codes: 0 ‘***’ 0.001 ‘**’ 0.01 ‘*’ 0.05 ‘.’ 0.1 ‘ ’ 1

exp(coef) exp(-coef) lower .95 upper .95

CCNB3 13.6678 0.07316 4.7850 39.0406

NEK4 0.6468 1.54609 0.5321 0.7862

ISY1 1.2598 0.79377 1.1249 1.4109

RIN2 1.0920 0.91574 1.0524 1.1332

POLG 1.1074 0.90302 1.0563 1.1610

TPM1 0.9746 1.02608 0.9569 0.9926

CD36 1.1374 0.87917 1.0472 1.2354

Concordance= 0.746 (se = 0.026 )

Likelihood ratio test= 72.82 on 7 df, p=4e-13

Wald test = 81.81 on 7 df, p=6e-15

Score (logrank) test = 88.77 on 7 df, p=<2e-16

--------------- Variance Inflating Factor (VIF) ---------------

Multicollinearity Problem: Variance Inflating Factor (VIF) is bigger than 10 (Continuous Variable) or is bigger than 2.5 (Categorical Variable)

CCNB3 NEK4 ISY1 RIN2 POLG TPM1 CD36

1.080636 1.159963 1.105970 1.101667 1.215749 1.134171 1.050966

# --------------------------------------------------------------------------------------------------

**### iter num = 7, Forward Selection by LR Test: + `MC1R`**

coef exp(coef) se(coef) z Pr(>|z|)

CCNB3 2.653422 14.202551 0.538701 4.926 8.41e-07 ***

NEK4 -0.471989 0.623761 0.101693 -4.641 3.46e-06 ***

ISY1 0.205348 1.227952 0.057809 3.552 0.000382 ***

RIN2 0.097318 1.102211 0.019343 5.031 4.87e-07 ***

POLG 0.104886 1.110584 0.024430 4.293 1.76e-05 ***

TPM1 -0.024124 0.976165 0.009286 -2.598 0.009378 **

CD36 0.139567 1.149775 0.042226 3.305 0.000949 ***

MC1R 0.026503 1.026858 0.007420 3.572 0.000354 ***

---

Signif. codes: 0 ‘***’ 0.001 ‘**’ 0.01 ‘*’ 0.05 ‘.’ 0.1 ‘ ’ 1

exp(coef) exp(-coef) lower .95 upper .95

CCNB3 14.2026 0.07041 4.9411 40.8234

NEK4 0.6238 1.60318 0.5110 0.7613

ISY1 1.2280 0.81436 1.0964 1.3753

RIN2 1.1022 0.90727 1.0612 1.1448

POLG 1.1106 0.90043 1.0587 1.1651

TPM1 0.9762 1.02442 0.9586 0.9941

CD36 1.1498 0.86974 1.0584 1.2490

MC1R 1.0269 0.97384 1.0120 1.0419

Concordance= 0.764 (se = 0.025 )

Likelihood ratio test= 79.27 on 8 df, p=7e-14

Wald test = 88.22 on 8 df, p=1e-15

Score (logrank) test = 98.78 on 8 df, p=<2e-16

--------------- Variance Inflating Factor (VIF) ---------------

Multicollinearity Problem: Variance Inflating Factor (VIF) is bigger than 10 (Continuous Variable) or is bigger than 2.5 (Categorical Variable)

CCNB3 NEK4 ISY1 RIN2 POLG TPM1 CD36 MC1R

1.080203 1.232940 1.243454 1.154388 1.229415 1.128835 1.054878 1.290115

# --------------------------------------------------------------------------------------------------

**### iter num = 8, Forward Selection by LR Test: + `SMC1B`**

coef exp(coef) se(coef) z Pr(>|z|)

CCNB3 2.690460 14.738454 0.540181 4.981 6.34e-07 ***

NEK4 -0.508999 0.601097 0.101187 -5.030 4.90e-07 ***

ISY1 0.209362 1.232891 0.057509 3.641 0.000272 ***

RIN2 0.098154 1.103132 0.019682 4.987 6.13e-07 ***

POLG 0.101346 1.106659 0.024518 4.133 3.57e-05 ***

TPM1 -0.022556 0.977696 0.009202 -2.451 0.014240 *

CD36 0.142386 1.153022 0.042292 3.367 0.000761 ***

MC1R 0.027516 1.027898 0.007410 3.713 0.000205 ***

SMC1B 0.422307 1.525477 0.138652 3.046 0.002321 **

---

Signif. codes: 0 ‘***’ 0.001 ‘**’ 0.01 ‘*’ 0.05 ‘.’ 0.1 ‘ ’ 1

exp(coef) exp(-coef) lower .95 upper .95

CCNB3 14.7385 0.06785 5.1127 42.4869

NEK4 0.6011 1.66362 0.4930 0.7330

ISY1 1.2329 0.81110 1.1015 1.3800

RIN2 1.1031 0.90651 1.0614 1.1465

POLG 1.1067 0.90362 1.0547 1.1611

TPM1 0.9777 1.02281 0.9602 0.9955

CD36 1.1530 0.86729 1.0613 1.2527

MC1R 1.0279 0.97286 1.0131 1.0429

SMC1B 1.5255 0.65553 1.1625 2.0018

Concordance= 0.77 (se = 0.025 )

Likelihood ratio test= 85.6 on 9 df, p=1e-14

Wald test = 95.62 on 9 df, p=<2e-16

Score (logrank) test = 108.4 on 9 df, p=<2e-16

--------------- Variance Inflating Factor (VIF) ---------------

Multicollinearity Problem: Variance Inflating Factor (VIF) is bigger than 10 (Continuous Variable) or is bigger than 2.5 (Categorical Variable)

CCNB3 NEK4 ISY1 RIN2 POLG TPM1 CD36 MC1R SMC1B

1.081458 1.277466 1.245385 1.162177 1.213885 1.137343 1.057807 1.307995 1.048497

# --------------------------------------------------------------------------------------------------

**### iter num = 9, Forward Selection by LR Test: + `LSP1P4`**

coef exp(coef) se(coef) z Pr(>|z|)

CCNB3 2.766931 15.909725 0.538481 5.138 2.77e-07 ***

NEK4 -0.482119 0.617474 0.100239 -4.810 1.51e-06 ***

ISY1 0.178076 1.194917 0.058247 3.057 0.002234 **

RIN2 0.099289 1.104386 0.020097 4.940 7.80e-07 ***

POLG 0.114113 1.120878 0.024954 4.573 4.81e-06 ***

TPM1 -0.026850 0.973507 0.009538 -2.815 0.004878 **

CD36 0.147965 1.159472 0.042751 3.461 0.000538 ***

MC1R 0.030183 1.030643 0.007610 3.966 7.30e-05 ***

SMC1B 0.432342 1.540863 0.136518 3.167 0.001541 **

LSP1P4 0.378343 1.459863 0.143934 2.629 0.008574 **

---

Signif. codes: 0 ‘***’ 0.001 ‘**’ 0.01 ‘*’ 0.05 ‘.’ 0.1 ‘ ’ 1

exp(coef) exp(-coef) lower .95 upper .95

CCNB3 15.9097 0.06285 5.5374 45.7108

NEK4 0.6175 1.61950 0.5073 0.7515

ISY1 1.1949 0.83688 1.0660 1.3394

RIN2 1.1044 0.90548 1.0617 1.1488

POLG 1.1209 0.89216 1.0674 1.1771

TPM1 0.9735 1.02721 0.9555 0.9919

CD36 1.1595 0.86246 1.0663 1.2608

MC1R 1.0306 0.97027 1.0154 1.0461

SMC1B 1.5409 0.64899 1.1791 2.0136

LSP1P4 1.4599 0.68500 1.1010 1.9357

Concordance= 0.791 (se = 0.022 )

Likelihood ratio test= 91.97 on 10 df, p=2e-15

Wald test = 101.1 on 10 df, p=<2e-16

Score (logrank) test = 114 on 10 df, p=<2e-16

--------------- Variance Inflating Factor (VIF) ---------------

Multicollinearity Problem: Variance Inflating Factor (VIF) is bigger than 10 (Continuous Variable) or is bigger than 2.5 (Categorical Variable)

CCNB3 NEK4 ISY1 RIN2 POLG TPM1 CD36 MC1R SMC1B

1.097747 1.278378 1.243569 1.160392 1.269938 1.155645 1.059767 1.289070 1.063921

LSP1P4

1.127458

# --------------------------------------------------------------------------------------------------

**### iter num = 10, Forward Selection by LR Test: + `ELL3`**

coef exp(coef) se(coef) z Pr(>|z|)

CCNB3 2.662453 14.331395 0.540061 4.930 8.23e-07 ***

NEK4 -0.496424 0.608704 0.100357 -4.947 7.55e-07 ***

ISY1 0.169224 1.184385 0.058454 2.895 0.00379 **

RIN2 0.096363 1.101159 0.020549 4.690 2.74e-06 ***

POLG 0.099102 1.104179 0.025358 3.908 9.30e-05 ***

TPM1 -0.025338 0.974980 0.009479 -2.673 0.00752 **

CD36 0.149516 1.161273 0.043148 3.465 0.00053 ***

MC1R 0.030952 1.031436 0.007749 3.995 6.48e-05 ***

SMC1B 0.387713 1.473606 0.136788 2.834 0.00459 **

LSP1P4 0.375105 1.455144 0.142822 2.626 0.00863 **

ELL3 0.150330 1.162217 0.067168 2.238 0.02521 *

---

Signif. codes: 0 ‘***’ 0.001 ‘**’ 0.01 ‘*’ 0.05 ‘.’ 0.1 ‘ ’ 1

exp(coef) exp(-coef) lower .95 upper .95

CCNB3 14.3314 0.06978 4.973 41.3037

NEK4 0.6087 1.64284 0.500 0.7410

ISY1 1.1844 0.84432 1.056 1.3282

RIN2 1.1012 0.90813 1.058 1.1464

POLG 1.1042 0.90565 1.051 1.1604

TPM1 0.9750 1.02566 0.957 0.9933

CD36 1.1613 0.86112 1.067 1.2638

MC1R 1.0314 0.96952 1.016 1.0472

SMC1B 1.4736 0.67861 1.127 1.9267

LSP1P4 1.4551 0.68722 1.100 1.9252

ELL3 1.1622 0.86042 1.019 1.3257

Concordance= 0.82 (se = 0.022 )

Likelihood ratio test= 96.68 on 11 df, p=8e-16

Wald test = 107.6 on 11 df, p=<2e-16

Score (logrank) test = 121.7 on 11 df, p=<2e-16

--------------- Variance Inflating Factor (VIF) ---------------

Multicollinearity Problem: Variance Inflating Factor (VIF) is bigger than 10 (Continuous Variable) or is bigger than 2.5 (Categorical Variable)

CCNB3 NEK4 ISY1 RIN2 POLG TPM1 CD36 MC1R SMC1B

1.091256 1.297069 1.243995 1.163898 1.287520 1.147105 1.059719 1.291350 1.070895

LSP1P4 ELL3

1.124477 1.093971

# --------------------------------------------------------------------------------------------------

**### iter num = 11, Forward Selection by LR Test: + `CDC25C`**

coef exp(coef) se(coef) z Pr(>|z|)

CCNB3 2.430679 11.366598 0.552990 4.396 1.11e-05 ***

NEK4 -0.431042 0.649831 0.102041 -4.224 2.40e-05 ***

ISY1 0.160846 1.174504 0.058624 2.744 0.006075 **

RIN2 0.101499 1.106828 0.020481 4.956 7.20e-07 ***

POLG 0.116475 1.123530 0.026548 4.387 1.15e-05 ***

TPM1 -0.026097 0.974241 0.009523 -2.740 0.006135 **

CD36 0.164441 1.178734 0.043013 3.823 0.000132 ***

MC1R 0.031431 1.031931 0.007956 3.951 7.79e-05 ***

SMC1B 0.345545 1.412759 0.139853 2.471 0.013482 *

LSP1P4 0.478059 1.612940 0.147429 3.243 0.001184 **

ELL3 0.170439 1.185825 0.065627 2.597 0.009402 **

CDC25C -0.008425 0.991610 0.003728 -2.260 0.023845 *

---

Signif. codes: 0 ‘***’ 0.001 ‘**’ 0.01 ‘*’ 0.05 ‘.’ 0.1 ‘ ’ 1

exp(coef) exp(-coef) lower .95 upper .95

CCNB3 11.3666 0.08798 3.8452 33.5998

NEK4 0.6498 1.53886 0.5320 0.7937

ISY1 1.1745 0.85142 1.0470 1.3175

RIN2 1.1068 0.90348 1.0633 1.1522

POLG 1.1235 0.89005 1.0666 1.1835

TPM1 0.9742 1.02644 0.9562 0.9926

CD36 1.1787 0.84837 1.0834 1.2824

MC1R 1.0319 0.96906 1.0160 1.0481

SMC1B 1.4128 0.70783 1.0741 1.8583

LSP1P4 1.6129 0.61999 1.2082 2.1533

ELL3 1.1858 0.84329 1.0427 1.3486

CDC25C 0.9916 1.00846 0.9844 0.9989

Concordance= 0.84 (se = 0.021 )

Likelihood ratio test= 102.3 on 12 df, p=<2e-16

Wald test = 109.5 on 12 df, p=<2e-16

Score (logrank) test = 124.3 on 12 df, p=<2e-16

--------------- Variance Inflating Factor (VIF) ---------------

Multicollinearity Problem: Variance Inflating Factor (VIF) is bigger than 10 (Continuous Variable) or is bigger than 2.5 (Categorical Variable)

CCNB3 NEK4 ISY1 RIN2 POLG TPM1 CD36 MC1R SMC1B

1.105764 1.363387 1.230375 1.195518 1.308840 1.151255 1.064727 1.286461 1.086197

LSP1P4 ELL3 CDC25C

1.163991 1.123395 1.194844

# ==================================================================================================

***** Stepwise Final Model (in.lr.test: sle = 0.05; out.lr.test: sls = 0.05; variable selection restrict in vif = 999999):**

Call:

coxph(formula = Surv(OS.time, OS.status) ~ CCNB3 + NEK4 + ISY1 +

RIN2 + POLG + TPM1 + CD36 + MC1R + SMC1B + LSP1P4 + ELL3 +

CDC25C, data = data, method = "efron")

n= 439, number of events= 93

coef exp(coef) se(coef) z Pr(>|z|)

CCNB3 2.430679 11.366598 0.552990 4.396 1.11e-05 ***

NEK4 -0.431042 0.649831 0.102041 -4.224 2.40e-05 ***

ISY1 0.160846 1.174504 0.058624 2.744 0.006075 **

RIN2 0.101499 1.106828 0.020481 4.956 7.20e-07 ***

POLG 0.116475 1.123530 0.026548 4.387 1.15e-05 ***

TPM1 -0.026097 0.974241 0.009523 -2.740 0.006135 **

CD36 0.164441 1.178734 0.043013 3.823 0.000132 ***

MC1R 0.031431 1.031931 0.007956 3.951 7.79e-05 ***

SMC1B 0.345545 1.412759 0.139853 2.471 0.013482 *

LSP1P4 0.478059 1.612940 0.147429 3.243 0.001184 **

ELL3 0.170439 1.185825 0.065627 2.597 0.009402 **

CDC25C -0.008425 0.991610 0.003728 -2.260 0.023845 *

---

Signif. codes: 0 ‘***’ 0.001 ‘**’ 0.01 ‘*’ 0.05 ‘.’ 0.1 ‘ ’ 1

exp(coef) exp(-coef) lower .95 upper .95

CCNB3 11.3666 0.08798 3.8452 33.5998

NEK4 0.6498 1.53886 0.5320 0.7937

ISY1 1.1745 0.85142 1.0470 1.3175

RIN2 1.1068 0.90348 1.0633 1.1522

POLG 1.1235 0.89005 1.0666 1.1835

TPM1 0.9742 1.02644 0.9562 0.9926

CD36 1.1787 0.84837 1.0834 1.2824

MC1R 1.0319 0.96906 1.0160 1.0481

SMC1B 1.4128 0.70783 1.0741 1.8583

LSP1P4 1.6129 0.61999 1.2082 2.1533

ELL3 1.1858 0.84329 1.0427 1.3486

CDC25C 0.9916 1.00846 0.9844 0.9989

Concordance= 0.85 (se = 0.021 )

Likelihood ratio test= 102.3 on 12 df, p=<2e-16

Wald test = 109.5 on 12 df, p=<2e-16

Score (logrank) test = 124.3 on 12 df, p=<2e-16

--------------- Variance Inflating Factor (VIF) ---------------

Multicollinearity Problem: Variance Inflating Factor (VIF) is bigger than 10 (Continuous Variable) or is bigger than 2.5 (Categorical Variable)

CCNB3 NEK4 ISY1 RIN2 POLG TPM1 CD36 MC1R SMC1B

1.105764 1.363387 1.230375 1.195518 1.308840 1.151255 1.064727 1.286461 1.086197

LSP1P4 ELL3 CDC25C

1.163991 1.123395 1.194844
