# Supplementary figures and images for: Identification and Validation of a Novel DNA Damage and DNA Repair Related Genes Based Signature for Colon Cancer Prognosis
Source: Front Genet. 2021 Feb 24;12:635863. doi: 10.3389/fgene.2021.635863 (PMC7943631; doi:10.3389/fgene.2021.635863)

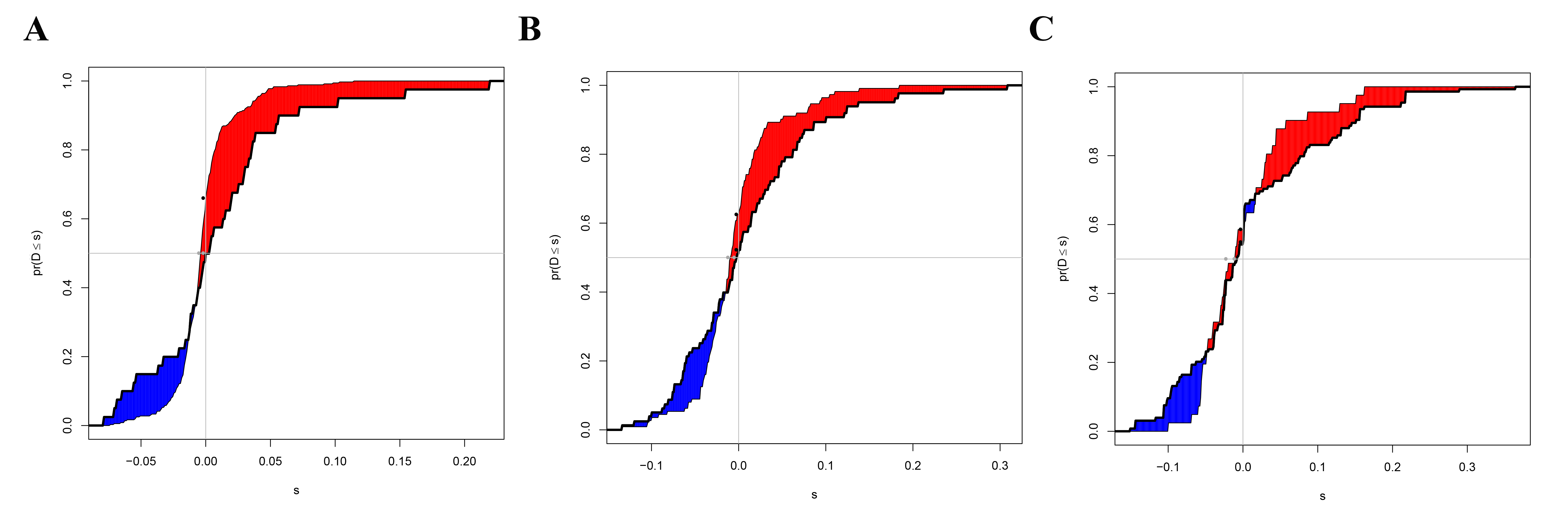

Supplement: Supplementary Figure 1 — The NRI and IDI analysis results for the 1year, 3 year, and 5 years survival prediction using a 12 gene model and 11 gene model. (A) 1 year, (B) 3 year, and (C) 5 year. [file Image_1.tif]
